# Prognostic scoring system based on indicators reflecting the tumor glandular differentiation and microenvironment for patients with colorectal cancer

**DOI:** 10.1038/s41598-024-65015-2

**Published:** 2024-06-20

**Authors:** Toshinori Kobayashi, Mitsuaki Ishida, Hisanori Miki, Nobuyuki Yamamoto, Takashi Harino, Takuki Yagyu, Soshi Hori, Masahiko Hatta, Yuki Hashimoto, Masaya Kotsuka, Makoto Yamasaki, Kentaro Inoue, Yoshinobu Hirose, Mitsugu Sekimoto

**Affiliations:** 1 Department of Surgery, Kansai Medical University, 2-5-1, Shinmachi, Hirakata City, Osaka 573-1010 Japan; 2 Department of Pathology, Osaka Medical and Pharmaceutical University, 2-7, Daigaku-Machi, Takatsuki City, Osaka 569-8686 Japan

**Keywords:** Cancer, Microbiology, Medical research, Oncology

## Abstract

Prognostic stratification is an urgent concern for patients with colorectal cancer (CRC). The desmoplastic reaction (DR) is speculated to mirror the tumor microenvironment. DR types are considered independent prognostic indicators in CRC, but have not been incorporated in previous prognostic nomograms. We aimed to assess the prognostic significance of a novel approach incorporating histopathological indicators reflecting tumor glandular differentiation and microenvironment. We evaluated 329 consecutive patients with CRC who underwent surgical resection at Kansai Medical University. Histological glandular differentiation was scored as 2 (0 point), 3 (1 point), or 4 (2 points). Tumor buddings (TBs) were classified as TB1 (0 point), TB2 (1 point), or TB3 (2 points). pT1 or 2 was considered as 0 point, pT3 or 4 + DR non-immature type as 1 point, and pT3 or 4 + DR immature type as 2 points. Lymph node metastasis was classified as pN0 (0 point), pN1 (1 point), or pN2 (2 points). The preoperative carcinoembryonic antigen levels were categorized as < 5.0 ng/mL (0 point) and ≧5.0 (1 point). Considering these factors, the following D&M (tumor differentiation and microenvironment) scoring system was applied: I (0–2 points), II (3–4 points), III (5–6 points), and IV (7–9 points). Kaplan–Meier curves showed significant differences in disease-specific survival and recurrence-free survival among the assigned scores, highlighting their enhanced utility compared with the American Joint Committee on Cancer 8th edition staging system. The D&M scoring system was valuable as the initial prognostic nomogram, including DR.

## Introduction

Establishing an effective prognostic stratification for patients with colorectal cancer (CRC) is a critical concern in the field of oncology, considering that CRC is the fourth leading cause of carcinoma, and its incidence is continuously increasing^1^. Various prognostic indicators and nomograms have been proposed^2–8^. The histopathological features of resected specimens offer crucial prognosis-related information. Traditionally, tumor differentiation (grading), determined based on the degree of glandular formation of tumor cells, has been recognized as a valuable prognostic indicator^9^; thus, it is included in some nomograms for patients with CRC^3,4^. Tumor differentiation is usually classified as well-, moderately, and poorly differentiated, or low-grade (well- and moderately differentiated) and high-grade (poorly differentiated)^9^. The combination of tumor differentiation grades (such as the sum of the primary and secondary predominant differentiation grades) demonstrated prognostic significance in patients with prostate cancer (Gleason grading system)^10^. Although the prognostic relevance of these histological glandular differentiation grading scores (HGDSs) has been reported in pancreatic and extrahepatic bile duct carcinomas^11,12^, their significance in patients with CRC remains unexplored. Moreover, tumor buddings (TBs), defined as a single tumor cell or a cluster of up to four tumor cells at the invasive front, is also an established histopathological prognostic indicator of CRC^13^ and other carcinoma types^14–16^.

The tumor microenvironment plays an important role in tumor invasion and metastasis^17^. Desmoplastic reaction (DR) refers to the fibrous tissue reaction around the tumor nests and is observed at varying degrees around the carcinoma cell nests. The prognostic significance of DR has been reported in CRC for the first time, highlighting DR type as a strong prognostic indicator of pT3 or pT4 CRC^18,19^ and other carcinoma types^16,20,21^. The type of DR is believed to reflect the tumor microenvironment and significantly influence prognosis, especially in stage II CRC patients^19,22,23^. Moreover, a recent study showed that automated detection using a deep learning system was useful for classifying DR and had superior prognostic significance^24^. Although the recently proposed nomogram calculates the risk of predicting recurrence following the resection of stage I–III colon cancer, including tumor-infiltrating lymphocytes (a crucial factor of the tumor microenvironment) as an indicator of recurrence^2^, no prognostic scoring system that incorporates DR has been established for CRC. Accordingly, the present study aimed to analyze the prognostic significance of combined histopathological indicators, considering both tumor glandular differentiation and microenvironment, which are easily obtained from routine clinical practice.

## Results

### Patient characteristics

Table 1 summarizes the clinical data of the present cohort. This study included 162 (49%) women and 167 (51%) men. The median age at the time of surgery was 72 years (range: 40–96 years). The tumor locations were as follows: right-side colon in 196 patients (60%), left-side colon in 108 (33%), and upper rectum in 25 (8%). The median body mass index was 22.6 (interquartile range: 14.1–35.0 kg/m^2^). On preoperative examination, the median total protein level and albumin level were 6.9 (interquartile range: 4.1–10.7 mg/dl) and 4 (interquartile range: 1.8–7.2 mg/dl), respectively, while the median hemoglobin level was 12.1 (interquartile range: 4.1–17.7 mg/dl). The median preoperative serum CEA levels were 4.0 (range: 1.0–2944 ng/dL). The median observation period from surgery was 48.9 (interquartile range: 0.27–79 months). Of these patients, 34 died due to causes unrelated to the disease. During the observation period, 52 patients experienced recurrences.

**Table 1:** Characteristics of the present cohort.

Variables	N = 329 (%)
Age, years, median [IQR]	72 [40–96]
Age ≥ 75 years	128 (38)
Sex, male/female	167 (51)/162(49)
Body mass index (kg/m^2^) [IQR]	22.6 [14.1–35.0]
Tumor location	
Right/left/rectum	196 (60)/108 (33)/25 (8)
Total protein (mg/dL) [IQR]	6.9 [4.1–10.7]
Albumin (mg/dL) [IQR]	4.0 [1.8–7.2]
Hemoglobin (mg/dL) [IQR]	12.1 [4.1–17.7]
Carcinoembryonic antigen (ng/dL) [IQR]	4.0 [1.0–2944]
Observation period (months) [IQR]	48.9 [0.27–79]

*IQR* interquartile range.

### Histopathological characteristics

Table 2 presents the clinicopathological features of the present cohort, excluding patients who died from causes other than CRC. Forty (14%), 33 (11%), 142 (48%), 48 (16%), and 32 (11%) patients had stages pT1, 2, 3, 4a, and 4b, respectively. Lymph node metastases occurred in 125 patients (42%). Sixty-four (22%), 96 (33%), 94 (31%), and 41 (14%) patients had pStages I, II, III, and IV, respectively.

**Table 2:** Clinicopathological features of the present cohort excluding the patients who died of causes other than colorectal carcinoma (N = 295).

Variables	Categories	(%)
pT	T1	40 (14)
	T2	33 (11)
	T3	142 (48)
	T4a	48 (16)
	T4b	32 (11)
pN	N0	170 (58)
	N1a	38 (13)
	N1b	33 (11)
	N1c	18 (6)
	N2a	23 (8)
	N2b	13 (4)
AJCC 8th TNM pStage	I	64 (22)
	II	96 (33)
	III	94 (31)
	IV	41 (14)

Table 3 presents a summary of the clinicopathological prognostic factors of the present cohort, excluding patients who died from causes other than CRC. The initial histological scores were 1 and 2 in 269 (92%) and 26 (9%) patients, respectively. The second histological scores were 1 and 2 in 229 (78%) and 66 (22%) patients, respectively. The HGDSs (the sum of 1st and 2nd histological scores) were 2, 3, and 4 in 211 (72%), 76 (25%), and 8 (3%) patients, respectively.

**Table 3:** Examination of clinicopathological prognostic factors for the patients in the present cohort, excluding those who died from causes unrelated to colorectal carcinoma (N = 295) and the trailing and validation cohorts.

Variables	Categories	N = 295 (%) Total	N = 197 (%) Training cohort	N = 98 (%) Validation cohort	P value
Histological glandular differentiation grading	2	211 (72)	*145 (74)*	*66 (67)*	*0.532*
	3	76 (25)	*47 (24)*	*29 (30)*	
	4	8 (3)	*5 (2)*	*3 (3)*	
Tumor budding grade	1	126 (43)	*87 (44)*	*39 (40)*	*0.674*
	2	53 (18)	*33 (17)*	*20 (20)*	
	3	116 (39)	*77 (39)*	*39 (40)*	
Desmoplastic reaction	pT1 + pT2	73 (25)	*51 (26)*	*22 (22)*	*0.811*
	pT3 or 4 + non-immature type	93 (32)	*61 (31)*	*32 (33)*	
	pT3 or 4 + immature type	129 (44)	*85 (43)*	*44 (45)*	
Lymph node metastasis	*pN0*	*170 (58)*	*111 (56)*	*59 (60)*	*0.322*
	*pN1*	*89 (29)*	*58 (28)*	*31 (32)*	
	*pN2*	*36 (13)*	*28 (14)*	*8 (8)*	
Carcinoembryonic antigen (ng/mL)	< 5	189 (64)	*124 (63)*	*65 (66)*	*0.568*
	≧5	106 (36)	*73 (37)*	*33 (34)*	
Prognostic scoring system	Score I	114 (39)	*76 (39)*	*38 (39)*	*0.9993*
	Score II	85 (29)	*56 (28)*	*29 (30)*	
	Score III	66 (22)	*45 (23)*	*21 (21)*	
	Score IV	30 (10)	*20 (10)*	*10 (10)*	

Significant values are in italic.

The TBs were categorized as 1, 2, and 3 in 126 (43%), 53 (18%), and 116 (39%) patients, respectively.

Non-immature and immature DRs of 93 and 129 pT3 and T4 patients, respectively. D&M (tumor differentiation and microenvironment) scoring system scores of I, II, III, and IV were assigned to 114 (39%), 85 (29%), 66 (29%), and 30 (10%) patients, respectively.

Table 3 shows all patients’ training and validation cohorts, as well as the clinicopathological background factors. There were no significant differences (p > 0.05) between HGDSs, TBs, DRs, and lymph node metastases, and the percentage of D&G scores was even.

### Cox hazard analysis of recurrence factors (D&G scale) for 254 patients with pStage I–III CRC according to AJCC 8th edition

Among the 254 patients, 52 experienced recurrence. The results of univariate analyses revealed significant variations (p < 0.05) across all D&G scoring factors. Multivariate analyses indicated a hazard ratio of 4.097 [1.233–13.604], a p-value of 0.0213 for HGDSs4, 13.353 [1.799–99.092], a p-value of 0.0113 for DR immature, and 2.740 [1.447–5.189], a p-value of 0.0020 for lymph node metastasis (Table 4).

**Table 4:** Cox hazard ratios of recurrence among the clinicopathological parameters in this study.

Univariate analysis	**Recurrence (N = 52)**	**P value**	Multivariate analysis	
Variable			Hazard ratio [95% CI]	P value
HGDSs 4	3	**0.00269**	**4.097 [1.233–13.604]**	**0.0213**
TB 3	33	** < 0.0001**	*1.823 [0.989–3.360]*	*0.0541*
DR immature	37	** < 0.0001**	**13.353 [1.799–99.092]**	**0.0113**
Lymph node metastasis	36	** < 0.0001**	**2.740 [1.447–5.189]**	**0.0020**
CEA ≧5	23	**0.0218**	*0.992 [0.564–1.762]*	*0.992*

*CEA* carcinoembryonic antigen *DR* desmoplastic reaction, *HGDSs* histological glandular differentiation grading scores, *TB* tumor budding.

Significant values are in bold and italic.

### Disease-specific survival analysis of 295 patients with CRC according to the AJCC 8th classification and D&M scoring system

**Figure 1:**
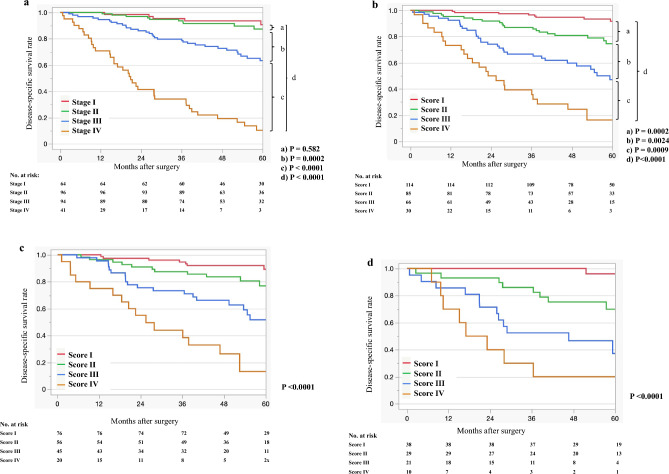
5 year disease-specific survival curves of patients with all stages of colorectal cancer (**a**) AJCC 8th edition staging system, (**b**) D&M scoring system, (**c**) training cohort using the D&M scoring system, and (**d**) validation cohort using the D&M scoring system.

Figure 1a shows the disease-specific survival (DSS) curves according to the currently used AJCC 8^th^ edition staging system^25^. The log-rank test showed no significant difference between stages I and II (P = 0.582). Significant differences were observed between stage II and stage III, stage III and stage IV, and among all stages (P = 0.0002, P < 0.0001, and P < 0.0001, respectively).

Figure 1b shows the DSS curves according to the D&M scoring system (scores I, II, III, and IV) in patients with all stages of CRC. Significant differences were observed between score I and score II, between score II and score III, and between score III and score IV, and among all scores in the DSS curves (P = 0.0002, P = 0.0024, and P = 0.0009, P < 0.0001, respectively).

The concordance index for DSS of the D&M scoring system was 0.71, and that of the AJCC 8th edition was 0.65.

### Disease-specific survival analysis of 295 patients with CRC in a training and validation cohort that stratified the D&M scoring system

Figures 1c and d show the DSS curves according to the D&M scoring system (scores I, II, III, and IV) in patients with all stages of CRC in the training and validation cohorts, respectively. A significant difference (P < 0.0001) in the DSS curves of the training and validation cohorts was observed.

### Recurrence-free survival analysis of 254 patients with CRC according to the AJCC 8th classification and D&M scoring system

**Figure 2:**
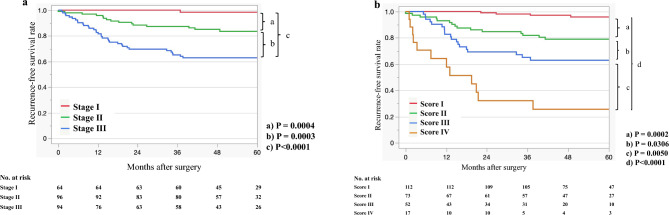
5 year recurrence-free survival curves of patients with pStage I, II, and III colorectal cancer. (**a**) AJCC 8th edition staging system. (**b**) D&M scoring system.

Figure 2a shows the recurrence-free survival (RFS) curves according to the AJCC 8th edition staging system^25^. Significant differences were observed between stage I and stage II, stage II and stage III, and among all stages (P = 0.0004 and P = 0.0003, P < 0.0001, respectively). Figure 2b shows the RFS curves according to the D&M scoring system (scores I, II, III, and IV) in patients with stage I–III CRC. Significant differences were observed between score I and score II, between score II and score III, between score III and score IV, and among all scores in the RFS curves (P = 0.0002, P = 0.0306, and P = 0.0050, P < 0.0001, respectively).

### Recurrence-free survival analysis of 94 patients with CRC (AJCC 8th pStage III) according to the AJCC 8th classification and D&M scoring system

**Figure 3:**
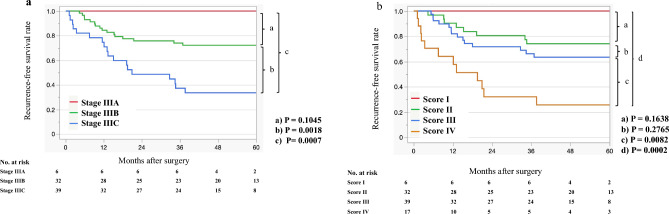
Recurrence-free survival curves of patients with pStage III colorectal cancer. (**a**) AJCC 8th edition staging system. (**b**) D&M scoring system.

Figure 3a shows the RFS curves according to the AJCC 8th edition staging system^25^. No significant difference was observed between stage IIIA and stage IIIB, but a significant difference was observed between stage IIIB and stage IIIC, and among all stages (P = 0.1045, P = 0.0018 and P = 0.0007, respectively). Figure 3b shows the RFS curves according to the D&M scoring system (scores I, II, III, and IV) in patients with stage III CRC. No significant difference was observed among scores I, II, and III, while a significant difference was found between scores III and IV and among all scores (P = 0.0082 and P = 0.0002, respectively).

## Discussion

The present study demonstrated the efficacy of the D&M scoring system in assessing the survival outcomes of patients with CRC. This prognostic scoring system is the first to combine histopathological indicators reflecting tumor glandular differentiation (HGDSs and TBs) and microenvironment (DR) in patients with CRC. Notably, this system exhibited a stronger correlation with DSS than the AJCC 8th edition staging system, especially in patients with pStages I, II, and III. Moreover, the D&M scoring system outperforms the conventional AJCC 8th edition staging system in predicting RFS. This superiority stems from its ability to effectively capture the prognostic significance of DR as a recurrence factor. This underscores the D&M scoring system’s capacity to provide enhanced insights into the recurrence risks of patients with CRC.

Prognostic stratification for patients with CRC has become a critical concern in the field of oncology, given the recent rise in the incidence of CRC and CRC-related death^1^. Thus, the development of a concise and convenient prognostic indicator for patients with CRC is an urgent concern to establish personalized treatment strategies for each patient with CRC based on the risk of recurrence^1^. The histopathological examination of the resected specimens provides essential information on the risk of recurrence and metastasis, such as depth of invasion, lymphovascular invasion, and lymph node metastasis, which serve as key indicators in the AJCC 8^th^ edition staging system^25^. However, the conventional AJCC staging system has survival paradoxes, particularly in patients with stage IIB/C and stage IIIA CRC^5^. In the present cohort, we observed that the conventional pStages I and II did not effectively predict DSS.

To address these survival paradoxes, various histological parameters and nomograms of patients with CRC have been proposed. The indicators used in the previously reported systems included tumor location, pT stage, lymph node metastasis status, histological grade, and neutrophil/lymphocyte ratio; however, some of these indicators were not applicable to all stages of CRC^3–7^. The Memorial Sloan Kettering Cancer Center nomogram, introduced in 2008 and revised in 2019, has been proposed for evaluating the risk of recurrence following curative surgical resection^26,27^. This nomogram incorporated nine clinicopathological factors, including age, serum CEA levels, T stage, lymph node metastasis, tumor location, lymphovascular and perineural invasion, and use of adjuvant chemotherapy^26,27^. Recently, the same group proposed a clinical calculator to evaluate the 3- and 5-year freedom from recurrence ratio of CRC based on the clinicopathological factors, including T stage, lymph node metastasis, use of postoperative chemotherapy, lymphovascular and perineural invasion, and tumor-infiltrative lymphocytes (TILs)^2^. This nomogram highlighted and reflected the prognostic significance of TILs^2^. However, no prognostic parameters have been established reflecting DR, which is recognized as a powerful prognostic indicator of CRC.

DR has garnered considerable attention and has been established as a useful independent prognostic indicator specifically in patients with pT3 or pT4 CRC^18,19^. DR is classified into three types: immature, intermediate, and mature. Notably, the immature-type DR exhibits a significantly worse prognosis compared with the intermediate and mature types^18,19^. The immature-type DR is significantly correlated with the presence of lymphovascular invasion, higher TB, and the presence of lymph node metastasis and tumor deposition^18,19,28^. The underlying mechanism behind the development of immature-type DR and its association with a poor prognosis have not yet been elucidated. However, carcinoma cells exhibit distinctive tissue reactions within the fatty tissue of the subserosal region (which is invaded only by pT3 or pT4 tumors). These unique tissue reactions occurring in the subserosal fatty tissue may serve as indicators of the prognosis of patients with CRC, with the tumor microenvironment speculated to play vital roles in DR development^19^. This prognostic scoring system is the first to incorporate DR as a prognostic indicator and significantly reflects the DSS.

**Figure 4:**
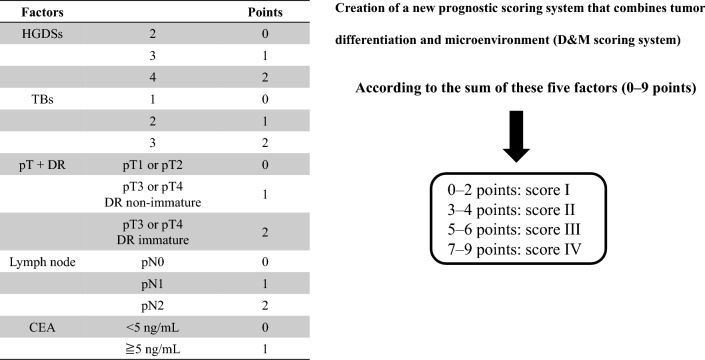
A schematic representation of the D&M scoring system based on the indicators reflecting tumor differentiation (HGDSs and TBs) and tumor microenvironment (DR) as well as depth of tumor invasion (pT) and lymph node metastasis (pN) and serum CEA levels. The sum of the five factors (0–9 points) was defined as scores I, II, III, and IV.

This prognostic scoring system for patients with CRC incorporated five clinicopathological indicators (Fig. 4). It included histopathological indicators reflecting tumor differentiation (HGDSs and TBs) and a combination of pT stage and DR (pT1 or pT2: 0 point, pT3 or pT4 + DR non-immature-type DR: 1 point, and pT3 or pT4 + immature-type DR: 2 points). Moreover, the lymph node metastatic status and preoperative serum CEA levels were included in this scoring system. pT stage and lymph node status were examined in all patients who underwent surgical resection for CRC. The simplicity of assessing HGDSs, TBs, and DR during routine histopathological tests using operative specimens of CRC, along with the routine evaluation of preoperative serum CEA levels in many hospitals, makes this grading system easily adaptable to daily clinical practice in treating CRC patients. Moreover, these indicators were significantly correlated with recurrence (Table 4), justifying their inclusion in the scoring system. Although the previously reported scoring system using HGDSs for patients with pancreatic and extrahepatic bile duct cancers adopted a three-tiered grading system (well-, moderately, and poorly differentiated as scores 1, 2, and 3, respectively), the present study used the simple two-tiered grading system (low and high grades as scores 1 and 2, respectively), consistent with the recent WHO classification^29^. Considering that DR was only defined in patients with pT3 and pT4, pT3 or pT4 + DR non-immature-type DR was assigned a score of 1 point, while pT3 or pT4 + immature-type DR was assigned a score of 2 points. Therefore, this grading system can be adopted to all CRC stages. Since the present study aimed to establish a prognostic scoring system reflecting the tumor glandular differentiation and tumor microenvironment, this scoring system does not include adjuvant chemotherapy as an indicator. This scoring system can be adopted in all CRC patients at the time of postoperative pathological diagnosis and provide prognostic information. The results of the present study demonstrated that this scoring system could reflect DSS and RFS in all stages of CRC, particularly in patients with pStages I, II, and III. The RFS analysis of patients with pStage III based on the AJCC 8th edition staging system was deemed inadequate due to the limited sample size. Therefore, further accumulation of cases is crucial to determine the efficacy between the pStages IIIA, B, and C, and the four classifications of the D&M scoring system established in the current study.

The present study has some limitations. First, this study was retrospective in nature and conducted in a single institute. Although this study included 329 patients with CRC, the presence of selection bias cannot be ruled out; that is, patients with pStages IIA, IIB, and IIC were classified as pStage II as well as pStage III. Second, the weighting of the histopathological factors and serum CEA levels requires further validation. For example, assigning a score of 2 points to both TB3 and pN2 raises questions about the significance of this finding. Therefore, a larger, multi-institutional study must be conducted to clarify this scoring system for patients with CRC.

We introduced a valuable prognostic scoring system that integrates histopathological indicators reflecting both tumor glandular differentiation and the microenvironment (D&M scoring system). This scoring system can effectively predict the prognosis in patients with all stages of CRC and can be easily evaluated through routine histopathological examination as well as the measurement of preoperative serum CEA levels. Additional multi-institutional studies with larger cohorts are required to validate the usefulness of this scoring system.

## Methods

### Patient selection

We selected 329 consecutive patients with CRC who underwent surgical resection at the Department of Surgery at Kansai Medical University Hospital between January 2016 and September 2019. Patients with low and middle rectal cancer were excluded as the majority of them received neoadjuvant chemotherapy and/or radiation therapy in our hospital. Moreover, two patients with neuroendocrine carcinoma were excluded as this type of carcinoma exhibits distinct clinicopathological features and poorer prognosis. The presence of distant metastases was examined using computed tomography. This study cohort partially overlapped with those of our previous study, which explored the relationship between the types of DR and tumor deposits. Tumor deposits are discrete macroscopic or microscopic nodules composed of carcinoma cells located in the extramural fatty tissue. They are discontinuous from the primary tumor and lack lymph node structures, serving as one of the poorest prognostic indicators of CRC^28^. However, the findings of this study differed from our previous research. Here, we analyzed the relationship between the type of DR and clinicopathological features, such as the presence of tumor deposits and TB. Notably, the prognostic significance of incorporating histopathological indicators reflect tumor glandular differentiation and the microenvironment^28^.

Eligible patients were divided into training and validation cohorts at a ratio of 7:3, and the training and validation cohorts comprised 197 and 98 participants, respectively.

### Histopathological analysis

Surgically resected specimens were fixed with formalin, sectioned, and stained with hematoxylin and eosin. Two researchers (Toshinori Kobayashi and Mitsuaki Ishida) independently evaluated the histopathological features of all tumor slides. The American Joint Committee on Cancer 8th Edition (AJCC 8th edition) staging system was employed^25^.

HGDSs were evaluated using the method used in the previous studies^11,12^. Histological grades were classified as low and high grades according to the recent World Health Organization (WHO) Classification^29^. Low grade (score 1) was defined as well-differentiated, characterized by well-formed tubular units with complete, easily discernible borders, and moderately differentiated, characterized by incomplete, ill-defined glandular borders, fusion of glands, or irregular multi-lumen cribriform pattern. High grade (score 2) was defined as poorly differentiated, characterized by non-glandular formation with cord-like, nested, solid, or individual cell infiltration. Mucinous adenocarcinomas were assigned a score of 2. By evaluating the whole tumor specimens, the primary and secondary predominant scores were determined for each case. The HGDSs were then evaluated by combining both scores (scores of 2–4). When the tumors comprised more than 90% of the same pattern, these cases were assigned the same score twice (for example, if the tumors comprised more than 90% of the low-grade component and 0–9% of the high-grade component, HGDS was assigned a score of 2).

The TBs were also evaluated using the same method as reported previously^30^. Tumors with 0–4 buds (defined as the presence of a single tumor cell or a cluster of up to four tumor cells) at the invasive front were classified as TB1, 5–9 buds as TB2, and more than 10 buds as TB3.

In patients with pT3 or 4 CRC, Ueno et al. classified DR into immature, intermediate, and mature types^18,19,31^. Briefly, the immature type is histopathologically characterized by the presence of myxoid stroma (defined as stroma accompanied by amorphous mucoid material), at a magnification of greater than 400 × , at the invasive front of the tumor. The intermediate type is characterized by the presence of keloid-like collagen (thick bundles of hypocellular collagen showing hyalinization) without a myxoid stroma. The mature type is marked by the absence of myxoid stroma and keloid-like collagen. Based on the specified definition, DR was not evaluated in pT1 and pT2 CRC.

Lymph node metastasis was evaluated according to the AJCC 8th edition staging system^25^. pN0 was defined as no lymph node metastasis, pN1 as metastasis in 1–3 lymph nodes (pN1a as metastasis in 1 regional lymph node, pN1b as metastasis in 2–3 regional lymph nodes, and pN1c as tumor deposit(s)), and pN2 as metastasis in four or more lymph nodes (pN2a as metastasis in 4–6 regional lymph nodes and pN2b as metastasis in seven or more regional lymph nodes)^25^.

### Creation of a new prognostic scoring system incorporating tumor differentiation and the tumor microenvironment (D&M scoring system)

The staging of patients with CRC was conducted according to the AJCC 8th edition staging system^25^, utilizing parameters such as the depth of tumor invasion (pT) and tumor lymph node and/or distant metastasis (pN and M). In addition to the existing AJCC 8th edition staging system, a new prognostic scale was devised based on the tumor differentiation grading (HGDSs and TBs) and the tumor microenvironment scoring (DR). The HGDSs were classified as 2 (0 point), 3 (1 point), and 4 (2 points). TBs were categorized as TB1 (0 points), TB2 (1 points), and TB3 (2 points). When pT and DR were combined, pT1 or 2 was scored as 0 point, pT3 or 4 + DR non-immature type (mature and intermediate types) as 1 point, and pT3 or 4 + DR immature type as 2 points. Lymph node metastasis was classified as pN0 (0 point), pN1 (1 point), or pN2 (2 points). The preoperative carcinoembryonic antigen (CEA) levels were classified as < 5.0 ng/mL (0 points) and ≧5.0 ng/mL (1 point). According to the sum of these five factors (0–9 points), scores of I (0–2), II (3–4), III (5–6), and IV (7–9) were assigned. This newly devised prognostic nomogram was defined as the D&M scoring system (Fig. 4). This scoring system includes known histopathological indicators that reflect tumor glandular differentiation and the microenvironment, T stage, lymph node status, and serum tumor markers. Although each indicator has been recognized to have prognostic significance, the present scoring system is the first to combine these markers in patients with CRC.

### Patient flowchart and endpoints

**Figure 5:**
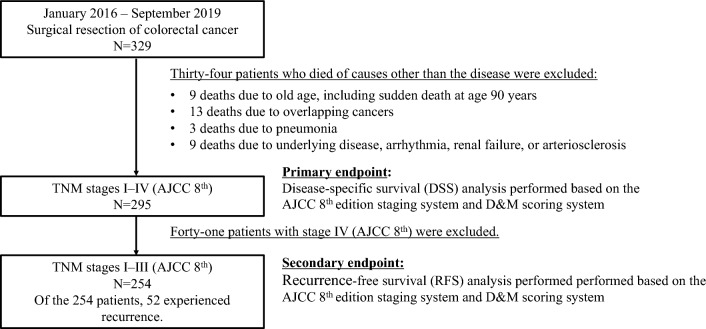
Algorithm depicting the study cohort.

During the study period, a total of 329 patients were initially considered. After excluding 34 patients who had died of other causes, only 295 were included in the prognostic evaluation. This study defined disease-specific survival (DSS) as the time from primary surgery to CRC-related death. In this context, the upper limit for the observation period was set at five years. The primary endpoint was DSS analysis in 295 patients using the AJCC 8th edition staging system and D&M scoring system. Recurrence-free survival (RFS) was defined as the period from the date of primary surgery to the detection of recurrence or the emergence of distant metastases. In this context, the upper limit for the observation period was set at five years. As a secondary endpoint, RFS was analyzed using the AJCC 8th edition staging system and the D&M score system in 254 patients with pStage I–III. Figure 5 shows a flowchart of the patient selection process.

This retrospective, single-institution study was conducted in accordance with the principles of the Declaration of Helsinki and was approved by the Institutional Review Board for Clinical Research of Kansai Medical University Hospital (approval number: 2021197). All data were anonymized. The Institutional Review Board for Clinical Research of Kansai Medical University Hospital waived the requirement for obtaining informed consent owing to the retrospective nature of the study; the utilization of the medical records and archived samples did not pose any risk to the participants. Moreover, this study did not include minors. Data regarding this study, such as the inclusion criteria and the option to opt-out, were provided on the institutional website (https://www.kmu.ac.jp/hirakata/hospital/2671t800001356c-att/a1642567101597.pdf).

Additionally, the preoperative serum CEA levels were assessed based on the information retrieved from the medical records.

### Statistical analyses

All analyses were performed using the JMP software version 17.0.0 (SAS Institute, Cary, NC, USA). The correlations between the two groups were analyzed using the chi-square test or Fisher’s exact test for categorical variables. The training and validation cohorts were randomly divided and assessed with D&G scores stratified at a ratio of 7:3. The Cox proportional hazards model was employed to assess recurrence risks in the D&M scales in patients with stages I-III (AJCC 8th edition) because this analysis aimed to determine the accurate clinicopathological parameters for predicting recurrence risk.

The DSS and RFS rates were compared using the Kaplan–Meier method, and P values were calculated using the log-rank test. The concordance index assessed the D&M scoring system and AJCC 8th edition staging system for their predictive capability. The concordance index was calculated using the area under the receiver operating characteristic curve (AUC), which plots the sensitivity against 1 minus the specificity of the nomogram. A P value of < 0.05 was considered significant.

## Data Availability

All data generated and analyzed in this study are included in this published article.
